# Functions of qualitative research and significance of the interpretivist paradigm in medical and medical education research

**DOI:** 10.20407/fmj.2020-018

**Published:** 2020-07-14

**Authors:** Takashi Otani

**Affiliations:** Graduate School of Education & Human Development, Nagoya University, Nagoya, Aichi, Japan

## What is Qualitative Research?

Scientific research in which researchers collect data comprises quantitative and qualitative approaches. The former collects quantitative data using measurements and analyzes these data statistically. The latter acquires qualitative linguistic data, such as interview and observation data, which are analyzed in various scientific ways. It is important to note that both approaches are empirical science.

The dominant focus of medical science and practice has been bio-medical. However, in 1977, Engel (1977) highlighted the necessity of a “bio-psycho-social” model.^1^ Since then, our society has also become more multi-/cross-cultural. Therefore, medical research is expected to include a psycho-socio-cultural aspect. Qualitative research can reflect this aspect, as it may investigate factors such as hope, belief, cognition, values, meaning, and the significance of human thoughts and conduct. These factors are subjective and inter-subjective, linguistic and non-linguistic, dynamic and interactional, irrational and contradictory, and are often not measurable quantitatively. In addition, a dominant aspect of education is essentially psycho-socio-cultural. Therefore, a qualitative approach has become indispensable in both medical research and medical education research; today, more qualitative research is conducted in these areas than before.

## Research Paradigms in Qualitative Research

**Figure 1:**
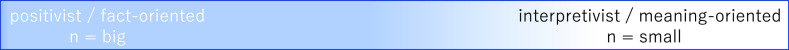
Spectrum of Qualitative Research

The paradigm in quantitative research is positivist without exception, meaning quantitative researchers can ignore the concept of a research paradigm. However, qualitative researchers adopt diverse approaches from very positivist to very interpretivist. Denzin and Lincoln (2017) discussed a comprehensive variety of research paradigms used in qualitative research.^2^ Otani (2019) indicated that these paradigms were continuously distributed within the “qualitative research spectrum” (Figure 1).^3^ This means that it is necessary for researchers to be aware of different research paradigms to fully understand qualitative research. This is particularly important as most qualitative medical research in Japan, with few exceptions such as Takahashi et al. (2018),^4^ has adopted a positivist paradigm rather than an interpretivist paradigm. This may partly be because such positivist qualitative research has an affinity with readers who are accustomed to quantitative research (which is completely positivist). However, it may also be because when qualitative research was introduced into Japan, a positivist paradigm was used. In other words, Japanese qualitative medical research has not yet caught up with international development of the paradigm shift in qualitative research. Therefore, a major challenge for medical qualitative research in Japan is to design and conduct qualitative research using an interpretivist paradigm, and demonstrate the meaningfulness of such an approach.

## The Qualitative Research Article in This Issue

Fortunately, this issue has one such article, titled “Moving beyond superficial communication to collaborative communication: learning processes and outcomes of interprofessional education in actual medical settings” by Mihoko Ito et al.^5^ This is a well-designed and well-conducted interpretivist qualitative study on medical interprofessional education. The authors’ interpretative analysis of interview data is excellent in its depth and significance, and their study offers a model for further qualitative medical and medical education research, both in Japan and internationally.

## Research Ethics in Qualitative Research

Research ethics is a crucial issue in qualitative research. For example, in quantitative research, it is possible to anonymize data to become unlinkable, so that even the researcher cannot know to whom the data belong. However, in qualitative research, although it is possible to preserve participant anonymity for readers, it is impossible to also make the data completely anonymous to the researcher. This is because qualitative data include information from which the researcher can identify a participant. This is just one example of how ethical issues differ between qualitative and quantitative research. In the abovementioned article, the authors designed, conducted, and reported their research carefully, meaning there were no ethical problems despite reaching a significant conclusion. Therefore, this article could also offer a model of research ethics of interpretative qualitative research.

## Expectation

I hope that the abovementioned article acts as a catalyst for the further development of medical and medical education qualitative research that uses a range of interpretative paradigms.
